# The prognostic and immune significance of C15orf48 in pan-cancer and its relationship with proliferation and apoptosis of thyroid carcinoma

**DOI:** 10.3389/fimmu.2023.1131870

**Published:** 2023-03-09

**Authors:** Chaolin Li, Yan Tang, Qin Li, Haiyan Liu, Xiaoying Ma, Liu He, Hao Shi

**Affiliations:** ^1^ Department of Obstetrics, Jinniu District Maternal and Child Health Hospital, Chengdu, China; ^2^ Department of Medical Laboratory, Jinniu District Maternal and Child Health Hospital, Chengdu, China; ^3^ Department of Pediatrics, Jinniu District Maternal and Child Health Hospital, Chengdu, China

**Keywords:** C15orf48, THCA, immunity therapy, apoptosis, biomarkers

## Abstract

**Background:**

C15orf48 was recently identified as an inflammatory response-related gene; however there is limited information on its function in tumors. In this study, we aimed to elucidate the function and potential mechanism of action of C15orf48 in cancer.

**Methods:**

We evaluated the pan-cancer expression, methylation, and mutation data of C15orf48 to analyze its clinical prognostic value. In addition, we explored the pan-cancer immunological characteristics of C15orf48, especially in thyroid cancer (THCA), by correlation analysis. Additionally, we conducted a THCA subtype analysis of C15orf48 to determine its subtype-specific expression and immunological characteristics. Lastly, we evaluated the effects of C15orf48 knockdown on the THCA cell line, BHT101, by *in vitro* experimentation.

**Results:**

The results of our study revealed that C15orf48 is differentially expressed in different cancer types and that it can serve as an independent prognostic factor for glioma. Additionally, we found that the epigenetic alterations of C15orf48 are highly heterogeneous in several cancers and that its aberrant methylation and copy number variation are associated with poor prognosis in multiple cancers. Immunoassays elucidated that C15orf48 was significantly associated with macrophage immune infiltration and multiple immune checkpoints in THCA, and was a potential biomarker for PTC. In addition, cell experiments showed that the knockdown of C15orf48 could reduce the proliferation, migration, and apoptosis abilities of THCA cells.

**Conclusions:**

The results of this study indicate that C15orf48 is a potential tumor prognostic biomarker and immunotherapy target, and plays an essential role in the proliferation, migration, and apoptosis of THCA cells.

## 1 Introduction

Cancer is a major public health concern worldwide (1). Studies show that approximately 3.21 million people died of cancer in 2022 (2). According to the latest assessment of the American Cancer Society, it is estimated that 609,820 people will die of cancer in the USA in 2023 (3). However, developments in immunotherapy, such as immune checkpoint (ICP)-targeting monoclonal antibodies and chimeric antigen receptor T cell therapy, have led to improvements in cancer treatment and prognosis (4, 5). Although these therapies have achieved great success in some cancers, such as breast cancer (BRCA) and glioblastoma (6, 7), their efficacy and post-treatment survival rates are low, especially for some metastatic cancers (8). Several studies have explored the common immunological features of cancers to determine the underlying mechanisms of tumorigenesis and progression (9); however, single cancer-targeting studies limit our understanding of the multifaceted nature of the cancer-related genes and features. Therefore, studies on the macroscopic ‘pan-cancer’ perspective might help reveal the underlying mechanism of tumorigenesis in malignant cancers (10–12).

C15orf48 (also known as Normal Mucosa of Esophagus-Specific Gene 1 protein [NMES1] and Modulator of Cytochrome C Oxidase during Inflammation [MOCCI]) was initially found to be downregulated in human esophageal squamous cell carcinoma (13), while another study found that it contributed to the development of colon cancer (14). C15orf48 forms a part of complex IV in the mitochondrial respiratory chain and interacts with multiple subunits in complexes I and IV (15, 16). Specifically, C15orf48 is a homolog of the NDUFA4 subunit of cytochrome C oxidase (complex IV), which replaces NDUFA4 in complex IV during inflammation, thereby reducing the membrane potential of mitochondria and reducing the production of reactive oxygen species (ROS), thus inhibiting immune response (17, 18). The inflammatory tumor microenvironment (TME) induced by chronic inflammation can greatly promote tumorigenesis (19). However, the potential role of C15orf48 has only been explored in esophageal squamous cell carcinoma and colon cancer, thus limiting the information on the role of C15orf48 in pan-cancer epigenetic changes, immunological characteristics, and prognosis. Therefore, in this study, we analyzed the pan-cancer expression level, methylation, single-cell mutation, copy number variation (CNV), and prognostic role of C15orf48. Additionally, by using multiple algorithms, we assessed the pan-cancer immunological signature of C15orf48 and its association with immunotherapy response. In addition, we analyzed the specific immunological characteristics, related functions, and subtype characteristics of C15orf48 in thyroid cancer (THCA) and verified the results by *in vitro* experimentation. The results of our study will help reveal the potential role of C15orf48 in tumor immunology and provide new directions for immunotherapy research.

## 2 Materials and methods

### 2.1 Data collection

The mRNA expression profiles and clinical data of 33 cancers were downloaded from the Cancer Genome Atlas (TCGA) database (https://portal.gdc.cancer.gov/), and the mRNA expression profiles of normal tissues were downloaded from the Genotype-Tissue Expression (GTEx) database (https://www.gtexportal.org/home/) and Human Protein Atlas (HPA) database (https://www.proteinatlas.org/). Cell line gene expression matrices for tumors were obtained from the Cancer Cell Line Encyclopedia dataset (CCLE, https://portals.broadinstitute.org/ccle/about). The CNV data of 11,495 samples were downloaded from the TCGA database and processed by Genomic Identification of Significant Targets in Cancer v2.0. We also downloaded the level 4 single nucleotide variation (SNV) dataset and Illumina HumanMethylation 450k level 3 data of all TCGA samples processed by MuTect2 (20). The glioblastoma dataset, CGGA325, was downloaded from the Chinese Glioma Genome Atlas (CGGA) database (http://www.cgga.org.cn/) (21). Lastly, tumor mutation burden (TMB) and microsatellite instability (MSI) data were derived from studies by Vesteinn Thorsson et al. and Russell Bonneville et al., respectively (22, 23). Abbreviations and sample information are provided in **Supplementary Table 1**.

### 2.2 Pan-cancer differential expression, prognosis, and epigenetic analysis of C15orf48

The HPA and GTEx data were used to analyze the expression of C15orf48 in the normal tissues. C15orf48 cancer cell line expression levels were analyzed using CCLE data, and C15orf48 single-cell expression was analyzed using HPA and Tumor Immune Single-cell Hub (TISCH) data (http://tisch.comp-genomics.org/). The expression profiles of TCGA and GTEx were integrated and the differential expression of C15orf48 in tumor and normal tissues was compared. Thereafter, the samples from 33 cancer types were divided into high- and low-expression groups according to the median expression of C15orf48. Thereafter, the R package “survival” was used to compare the survival time and survival status of the two groups. The *p*-values and hazard ratios (HR, with 95% confidence intervals [CI]), for the Kaplan–Meier curves, were derived by log-rank test and univariate cox regression analysis. The time-dependent receiver operating characteristic (timeROC) analysis was used to compare the prediction accuracy of C15orf48, while univariate and multivariate cox regression analyses were used to assess its value as an independent prognostic factor.

We assessed the C15orf48 methylation levels of normal and pan-cancer tumor tissues and divided the tumor samples into high- and low-methylation groups according to the median C15orf48 methylation level. Spearman correlation analysis was used to obtain the correlation between C15orf48 mRNA expression and methylation level. Thereafter, the R package “survival” was used to compare the survival time and survival status of the two groups. The pan-cancer C15orf48 SNV data were visualized using the R package “maftools”. In addition, we assessed the pan-cancer C15orf48 CNV data and its association with pan-cancer prognosis. We also assessed the correlation of C15orf48 with pan-cancer TMB and MSI and the correlation between C15orf48 and 44 marker genes of three classes of RNA modifications (m1A, m5C, and m6A).

### 2.3 Association between C15orf48 and pan-cancer immune cell infiltration and immunotherapy response

The stromal, immune, and ESTIMATE scores of each tumor sample were calculated according to C15orf48 expression, using the R package “Estimation of STromal and Immune cells in MAlignant Tumor tissues using Expression data” (ESTIMATE) v1.0.13 (24). We used 5 algorithms, including single-sample Gene Set Enrichment Analysis (ssGSEA), Cell-type Identification by Estimating Relative Subsets of RNA Transcripts (CIBERSORT), Tumor IMmune Estimation Resource (TIMER), Estimating the Proportion of Immune and Cancer cells (EPIC), and Microenvironment Cell Populations (MCP)-counter, to determine the correlation between C15orf48 and pan-cancer ICI. We also evaluated the response of C15orf48 high- and low-expression groups to programmed cell death protein 1 (PD-1) and cytotoxic T-lymphocyte associated protein 4 (CTLA4) immunotherapy (25) from the Cancer Immunome Atlas data (TCIA, https://tcia.at/home).

### 2.4 Immunological characteristics, functional enrichment, and subtype characteristics of C15orf48 in THCA

Based on a study by Charoentong et al. (25), we obtained 122 immune modulators and evaluated their correlation with C15orf48 mRNA expression in THCA. The anti-cancer immune state reflects the various activities of the cancer immune cycle. We used the Tracking Tumor Immunophenotype (TIP) database (http://biocc.hrbmu.edu.cn/TIP/) to assess the anti-cancer immune status at 7 different stages of the tumor immune cycle, including the release of cancer cell antigens (step 1), cancer antigen presentation (step 2), priming and activation (step 3), trafficking of immune cells to tumors (step 4), ICI in tumors (step 5), recognition of cancer cells by T cells (step 6), and killing of cancer cells (step 7) (26). We used 7 algorithms, including CIBERSORT under absolute mode (CIBERSORT-ABS), MCP-counter, quantification of the Tumor Immune contexture from human RNA-seq data (quantIseq), TIMER, xCell, EPIC, and Tumor-Immune System Interactions database (TISIDB, http://cis.hku.hk/TISIDB/index.php), to calculate the level of ICI of C15orf48 in THCA. The list of genes for the immune process was obtained from the AmiGO 2 portal (http://amigo.geneontology.org/amigo). The correlation between C15orf48 and the immune process was determined using the R package “Gene set variation analysis” (GSVA). In addition, we also calculated the correlation between immune cell marker genes and C15orf48 in THCA.

The Search Tool for Retrieval of Interacting Genes/Proteins (STRING) database (https://string-db.org/) was used to analyze the protein interaction network of C15orf48. The differential expression of C15orf48 high- and low-expression groups in THCA was studied using the R package “Limma” v3.40.2. Furthermore, the R package “ClusterProfiler” was used for Gene Ontology (GO) and Kyoto Encyclopedia of Genes and Genomes (KEGG) enrichment analyses. In addition, we collected the gene sets from the relevant pathways (27) and calculated the correlation between gene expression and pathways according to the ssGSEA algorithm. We also evaluated the expression level of C15orf48, the immune signature, and response to immunotherapy among different THCA subtypes, such as papillary thyroid carcinoma (PTC) and follicular thyroid carcinoma (FTC).

### 2.5 Cell culture, real-time quantitative reverse transcription PCR, and western blotting analyses

The human THCA cell line, BHT101, was purchased from Shanghai Jinyuan Biotechnology (Shanghai, China) and cultured in the indicated medium with 10% phosphate buffer saline (PBS). The cells were incubated at 37°C and 5% CO_2_. Total RNA was extracted with TRIzol reagent (Invitrogen, USA) and reverse transcribed with random primers using Hiscipt III 1st strand cDNA synthesis kit (Vazyme, Nanjing, China) according to the manufacturer’s instructions. The following primers were used for qRT-PCR: GAPDH forward primer: 3’-GGAGCGAGATCCCTCCAAAAT-5’, reverse primer: 3’-GGCTGTTGTCATACTTCTCATGG-5’ and C15orf48 forward primer: 3’-AACTCATTCCCTTGGTGGTGTTCAT-5’, reverse primer: 3’-CTCGTCATTTGGTCACCCTTTGGAC-5’.

The cells were transfected with C15orf48 siRNA, harvested, washed thrice with PBS, and collected by centrifugation. Total protein extracts were prepared in radioimmunoprecipitation assay (RIPA) buffer supplemented with proteinase inhibitors (R0010, Solarbio). Anti-C15orf48 (NBP1-98391, Novus Biologicals) and anti-GAPDH (60004-1-Ig, Proteintech) antibodies were used for western blot analysis according to the manufacturer’s instructions. Goat anti-mouse IgG-HRP (SA00001-1, Proteintech) and goat anti-rabbit IgG-HRP (SA00001-2, Proteintech) were used as secondary antibodies. GAPDH was used as a protein loading control. The signals were visualized using the enhanced chemiluminescence (ECL) reagent (4A Biotech, China).

### 2.6 Cell counting kit-8 analysis

BHT101 cells transfected with C15orf48 siRNA were digested once they reached 90% confluency and inoculated into 96-well culture plates at 5000 cells/well and 5 wells/group. Thereafter, the cells were cultured in a 37°C and 5% CO_2_ incubator and analyzed at 0, 24, 48, and 72 h using the CCK-8 kit (WLA074, China).

### 2.7 Wound healing test

BHT101 cells were inoculated in 6-well plates and transfected with C15orf48 siRNA. Thereafter, the cells were scraped with a 200 μl pipette tip. The cell surface was cleaned with a serum-free medium and the cell fragments were removed. The cells were then observed and photographed under a 40× microscope and their positions in the photos were recorded. Subsequently, cells in each group were placed in a 37°C and 5% CO_2_ incubator for 24 and 48 h, after which they were photographed and recorded. Lastly, the mobility of each group was calculated.

### 2.8 Transwell migration and apoptotic assay

A 24-well Transwell chamber (8 μm aperture; Corning Costar, USA) was prepared overnight at 4°C and inoculated with 200 μl of cell suspension containing 100,000 cells/mL. A culture medium (700 μl) containing 10% fetal bovine serum was poured into the lower chamber. After 24 h of incubation at 37°C and 5% CO_2_, the cells were fixed using 4% paraformaldehyde at room temperature for 20 mins, stained with 0.5% crystal violet dye for 5 mins, and the cell count was recorded.

BHT101 cells were harvested and resuspended in a binding buffer. Thereafter, the cells were stained with Annexin V-FITC/PI Apoptosis Detection kit (Vazyme, Nanjing, China) according to the manufacturer’s instructions. The cells were then analyzed by flow cytometry (Cytoflex, Beckman) and the data were analyzed using CytExpert Software.

### 2.9 Statistical analysis

All the analysis methods and R packages were implemented using R version 4.1.0, except for the online website tools. Wilcoxon rank-sum test was used to calculate differential expression in normal and tumor samples. Univariate cox regression analysis was done with the “forestplot” R package. We used the Spearman correlation method to perform correlation analysis between C15orf48 transcript levels and immune checkpoint gene expression, TMB levels, and MSI status. Data from cell experiments were analyzed using GraphPad Prism (version 9.0.0) for Windows. All the experiments were repeated in triplicate. Student’s t-test was used to assess statistical significance. P values less than 0.05 were considered statistically significant. *p < 0.05; **p < 0.01; ***p < 0.001.

## 3 Results

### 3.1 Pan-cancer expression of C15orf48

**Figure 1:**
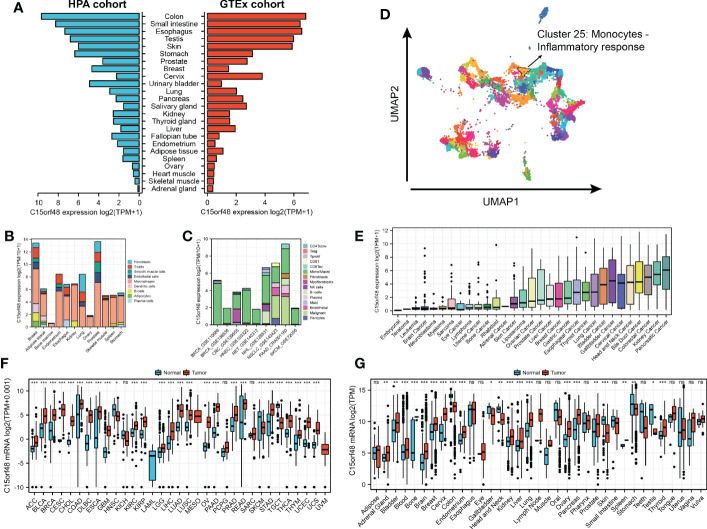
**(A)** Expression level of C15orf48 in normal tissues (HPA+GTEx datasets); **(B)** C15orf48 expression levels in single cells (HPA datasets); **(C)** C15orf48 expression levels in single cells (TISCH datasets); **(D)** C15orf48 is part of cluster 25 Monocytes - Inflammatory response; **(E)** Expression levels of C15orf48 in cancer cell lines (CCLE datasets); **(F)** Differences in the expression of C15orf48 between normal and cancerous tissues (TCGA+GTEx datasets); **(G)** Differences in the expression of C15orf48 between normal and cancerous tissues (GEO datasets). *p < 0.05; **p < 0.01; ***p < 0.001; NS, No Significance.

Analysis of the HPA and GTEx datasets revealed higher expression of C15orf48 in the colon, small intestine, esophagus, and other normal tissues (**Figure 1A**). Additionally, C15orf48 protein expression was significantly elevated in multiple cancers (**Supplementary Figures 1A, B**). Furthermore, the single-cell analysis revealed cell-specific expression of C15orf48. Analysis of the HPA single-cell dataset and TISCH online dataset revealed that C15orf48 was significantly overexpressed in macrophages (**Figures 1B, C**). Moreover, we observed a significant enrichment of C15orf48 in macrophages in some datasets that received immunotherapy (**Supplementary Figures 1C, D**). Furthermore, correlation analysis between C15orf48 expression and immune cell clustering revealed that C15orf48 is a part of cluster 25 monocytes—inflammatory response with confidence 1 (**Figure 1D**). Moreover, analysis of the cancer cell lines revealed high expression of C15orf48 in specific cancer types, such as pancreatic cancer, kidney cancer, and colorectal cancer (**Figure 1E**). Additionally, analysis of the integrated TCGA and GTEx data revealed a significantly high expression of C15orf48 in multiple cancers, including THCA (**Figure 1F**). These results were further validated by the pan-cancer C15orf48 expression data (platform: GPL570; HG-U133_Plus_2) obtained from the Gene Expression Omnibus (GEO) database (**Figure 1G**).

### 3.2 C15orf48 is an independent prognostic factor for glioma

**Figure 2:**
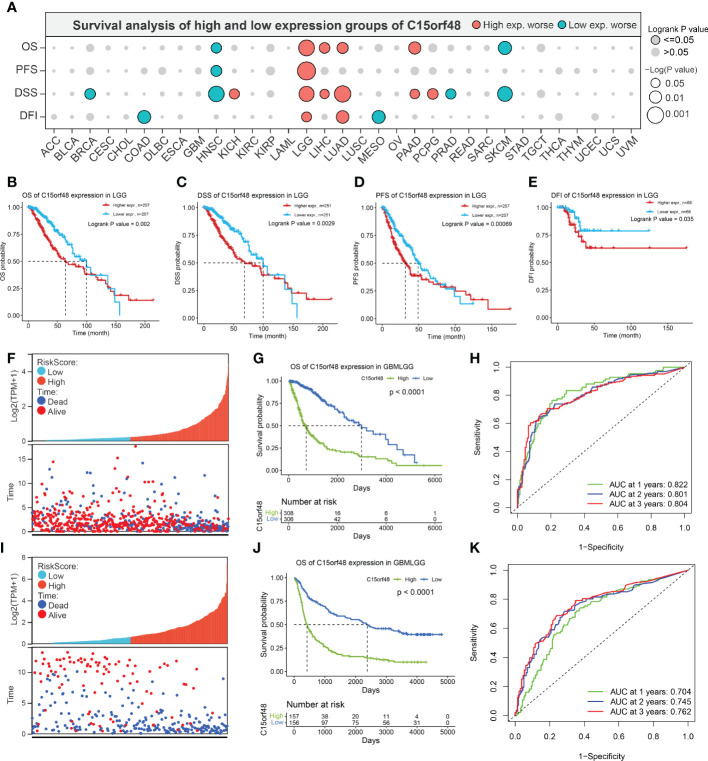
**(A)** Prognosis of C15orf48 in pan-cancer; **(B-E)** High expression of C15orf48 was significantly correlated with shorter OS, DSS, PFS, and DFI of LGG; **(F-H)** Relationship between C15orf48 expression and glioma prognosis score, survival analysis and ROC analysis (TCGA-GBMLGG datasets); **(I–K)** Relationship between C15orf48 expression and glioma prognosis score, survival analysis and ROC analysis (CGGA325 datasets).

Pan-cancer prognostic analysis revealed that C15orf48 was significantly associated with the prognosis of multiple cancers (**Figure 2A**). Specifically, high expression of C15orf48 was significantly associated with shorter overall survival (OS), progression-free survival (PFS), disease-specific survival (DSS), and disease-free interval (DFI) in low-grade gliomas (LGGs) (**Figures 2B–E**). Furthermore, high expression of C15orf48 was significantly associated with shorter OS and DSS in liver hepatocellular carcinoma, lung adenocarcinoma, and pancreatic adenocarcinoma (PAAD) (**Supplementary Figure 2A**). In addition, varying degrees of prognostic correlations were also observed in head and neck squamous cell carcinoma, skin cutaneous melanoma, BRCA, colon adenocarcinoma, mesothelioma, and prostate adenocarcinoma (PRAD) (**Supplementary Figure 2A**). Considering its significant association with glioma prognosis, we further evaluated the clinical significance of C15orf48 in glioma. The results showed that C15orf48 was significantly enriched in high-grade glioma, non-1p/19q deletion state, wild-type, and non- O (6)-methylguanine-DNA-methyltransferase (MGMT) promoter methylated samples in both TCGA and CGGA datasets (**Supplementary Figure 2B**). These results indicated that C15orf48 was highly enriched in more malignant gliomas. In addition, we combined the clinical and expression data of TCGA-glioblastoma multiforme (GBM) and TCGA-LGG and then evaluated the association between C15orf48 gene expression and patient survival time and survival status. The results showed that glioma patients with high C15orf48 expression had a significantly higher mortality rate with the 1-, 2-, and 3-y mortality prediction areas under the curve (AUC) values of 0.822, 0.801, and 0.804, respectively (**Figures 2F–H**). These results were further validated using the CGGA database (**Figures 2I–K**). Lastly, univariate and multivariate cox regression analysis of TCGA and CGGA datasets revealed that C15orf48 can serve as an independent prognostic factor for glioma (**Tables 1**, **2**).

**Table 1:** Univariate and multivariate analyzes of OS prognostic parameters in the TCGA database.

Variable	Univariate analysis		Multivariate analysis	
	HR (95% CI)	*p* value	HR (95% CI)	*p* value
C15orf48	1.501 (1.405-1.604)	2.28E-33	1.114 (1.000-1.241)	0.049
Age	5.043 (3.348-7.596)	9.82E-15	3.605 (2.273-5.716)	5.03E-08
WHO grade	9.544 (6.813-13.371)	2.70E-39	3.957 (2.398-6.530)	7.40E-08
1p/19q Codel	0.220 (0.130-0.375)	2.31E-08	0.397 (0.223-0.705)	0.002
MGMT status	0.312 (0.225-0.433)	2.96E-12	0.627 (0.433-0.909)	0.014

**Table 2:** Univariate and multivariate analyzes of OS prognostic parameters in the CGGA database.

Variable	Univariate analysis		Multivariate analysis		
	HR (95% CI)	*p* value	HR (95% CI)		*p* value
C15orf48	2.876 (2.089-3.958)	9.17E-11	1.438 (1.023-2.022)		0.036
Age	1.614 (1.214-2.145)	0.001	1.068 (0.791-1.440)		0.668
WHO grade	4.885 (3.634-6.566)	8.02E-26	3.082 (2.239-4.242)		5.08E-12
1p/19q Codel	0.170 (0.104-0.277)	1.25E-12	0.256 (0.154-0.426)		1.48E-07
MGMT status	0.830 (0.632-1.089)	0.178			

### 3.3 Pan-cancer epigenetic variations of C15orf48

**Figure 3:**
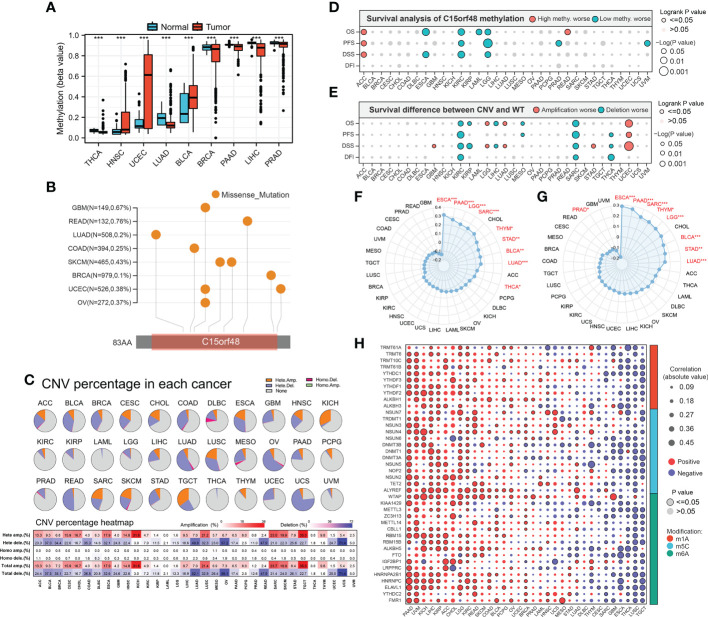
**(A)** Differential methylation levels of C15orf48 in normal and tumor tissues in pan-cancer; **(B)** Somatic mutation levels of C15orf48 in pan-cancer; **(C)** Copy number variation levels of C15orf48 in pan-cancer; **(D)** The relationship between methylation of C15orf48 and prognosis in pan-cancer; **(E)** The relationship between copy number variation of C15orf48 and prognosis in pan-cancer; **(F)** Relationship between C15orf48 and tumor mutational burden in pan-cancer; **(G)** Relationship between C15orf48 and microsatellite instability in pan-cancer; **(H)** Correlation between C15orf48 and RNA-modifying genes in pan-cancer. *p < 0.05; **p < 0.01; ***p < 0.001.

We further explored the methylation levels of C15orf48 to determine its epigenetic regulation. As shown in **Figure 3A**, C15orf48 exhibits differential methylation levels in various cancer and normal tissues. Furthermore, the methylation level of C15orf48 was negatively correlated with its mRNA expression to varying degrees in all cancers (**Supplementary Figure 3A**). Somatic mutations of C15orf48 were primarily missense mutations and the overall somatic mutation rate of C15orf48 was <1%, with the highest mutation rate in rectum adenocarcinoma (READ, 0.76%) (**Figure 3B**). The CNV of C15orf48 in different tumors was highly heterogeneous (**Figure 3C**), among which we analyzed both homozygous and heterozygous deletions and amplification. The results showed that heterozygous amplification was prevalent in kidney chromophobe (KICH) and testicular germ cell tumors (TGCT), while heterozygous deletion was prevalent in uterine carcinosarcoma, READ, LUAD, and ovarian serous cystadenocarcinoma. Moreover, the prognostic analysis showed that a high methylation level of C15orf48 was significantly associated with shorter OS, PFS, and DSS in adenoid cystic carcinoma, whereas, a low methylation level of C15orf48 was significantly associated with the poor prognosis of esophageal carcinoma (ESCA), kidney renal clear cell carcinoma (KIRC), acute myeloid leukemia, LGG, and PRAD (**Figure 3D**; **Supplementary Figure 3B**). In addition, deletion mutation of C15orf48 was significantly associated with poor prognosis of KIRC, sarcoma (SARC), and THCA, while amplification of C15orf48 was significantly associated with poor prognosis of LGG and uterine corpus endometrial carcinoma (**Figure 3E**; **Supplementary Figure 3C**). TMB and MSI are closely associated with clinical treatment and tumor markers. The expression of C15orf48 was significantly correlated with TMB in ESCA, PAAD, LGG, SARC, THCA, etc. (**Figure 3F**) and significantly correlated with MSI in ESCA, PAAD, SARC, LGG, etc. (**Figure 3G**). Furthermore, marker genes of C15orf48 and RNA modification showed different degrees of correlation in different cancers (**Figure 3H**).

### 3.4 Association between C15orf48 and pan-cancer ICI and immunotherapy response

**Figure 4:**
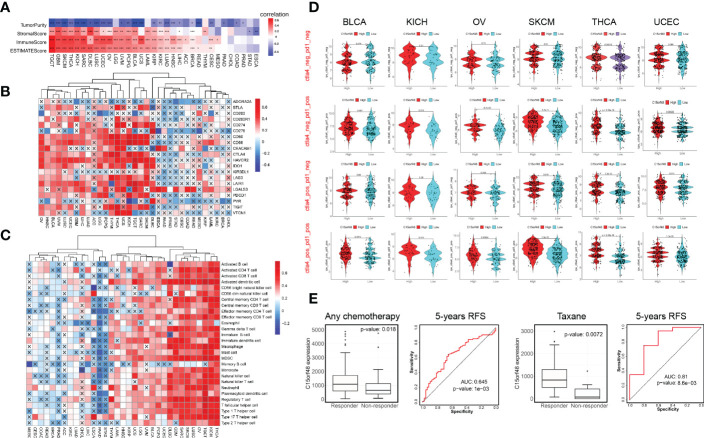
**(A)** Correlation between C15orf48 and tumor purity, stromal, immune, and ESTIMATE scores in pan-cancer; **(B)** Correlation between C15orf48 and immune checkpoints in pan-cancer; **(C)** Correlation between C15orf48 and immune cell infiltration in pan-cancer (ssGSEA); **(D)** Correlation between C15orf48 and immunotherapy response in pan-cancer; **(E)** Box plots show the C15orf48 expression differences between responders and non-responders, and ROC presents the predictive accuracy of patient therapeutic response by C15orf48 levels on the ROCplotter online website. *P < 0.05; **P < 0.01; ***P < 0.001.

C15orf48 has a strong positive correlation with immune cells and stromal cells in several cancers, including TGCT, GBM, THCA, etc. (**Figure 4A**) and with ICPs in TGCT, THCA, KICH, etc. (**Figure 4B**). Several algorithms, including ssGSEA (**Figure 4C**), CIBERSORT, TIMER, EPIC, and MCP-counter (**Supplementary Figures 4A–D**), were used to assess the association of C15orf48 with pan-cancer ICI, and the results revealed that C15orf48 is positively correlated to various levels of ICI in THCA, KICH, TGCT, etc. In addition, C15orf48 was significantly positively correlated with the infiltration scores of major histocompatibility complex (MHC) and effector cells in TGCT, THCA, and SARC, while it was significantly negatively correlated with the infiltration scores of ICPs and immunosuppressive cells (**Supplementary Figure 4E**).

Furthermore, we determined the effect of C15orf48 on pan-cancer immunotherapy response. Immunophenoscore (IPS) was used to evaluate the immunotherapy response between the C15orf48 high- and low-expression groups (25), and the results revealed that the C15orf48 high-expression group showed strong immunogenicity upon receiving PD-1, CTLA4, and combination therapy (**Figure 4D**). In addition, we further evaluated the predictive role of C15orf48 on cancer therapy response using the ROC Plotter database (https://www.rocplot.org/) (28), and the results revealed that C15orf48 was highly expressed in BRCA patients responding to chemotherapy and the AUC value of 5-y recurrence-free survival (RFS) reached 0.645. Moreover, in patients receiving taxane treatment, the AUC of 5-y RFS reached 0.81 (**Figure 4E**).

### 3.5 Immunological characteristics, functions, and subtype distribution of C15orf48 in THCA

**Figure 5:**
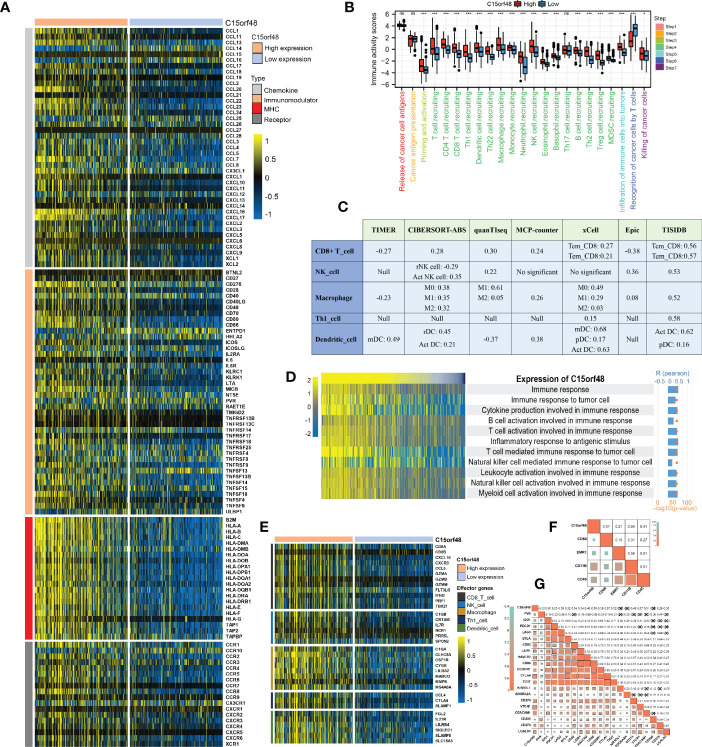
**(A)** Correlation between C15orf48 and 122 immune modulators (chemokines, receptors, MHC and immunostimulants) in THCA; **(B)** Differences in the various steps of the cancer immunity cycle between high- and low-C15orf48 groups; **(C)** Seven algorithms evaluate the correlation between C15orf48 and immune cell infiltration in THCA; **(D)** GSVA assesses the relevance of C15orf48 to some immune pathways; **(E)** Differences in the effector genes of the tumor-associated immune cells between high- and low-C15orf48 groups; **(F)** Correlation between C15orf48 and macrophage marker genes in THCA; **(G)** Correlation between immune checkpoints and C15orf48 in THCA. *p < 0.05; **p < 0.01; ***p < 0.001; NS, No Significance.

We observed a strong positive association between C15orf48 and multiple immune modulators (**Figure 5A**). Some key monocyte/macrophage chemokines (CCL7, CCL22, etc.) were upregulated in the C15orf48 high-expression group, promoting inflammatory response and monocyte/macrophage phagocytosis in THCA. Additionally, a large number of MHC molecules were significantly upregulated in THCA in the C15orf48 high-expression group, indicating strong antigen presentation and processing capabilities. Moreover, we observed that the C15orf48 high-expression group has a stronger anti-cancer immune status in most immune cycle steps, including priming and activation (step 3), trafficking of immune cells to tumors (step 4), ICI in tumors (step 5), and killing of cancer cells (step 7) (**Figure 5B**). The stronger immune status of the C15orf48 high-expression group may further enhance ICI in the THCA-TME. Furthermore, we observed that C15orf48 expression was negatively correlated with the recognition of cancer cells by T cells (step 6), suggesting that the high expression of C15orf48 may reduce the recognition-ability of T cell receptors. The analysis of ICI level showed that in most algorithms, C15orf48 was positively correlated with 5 types of ICIs, including CD8+ T cell, NK cell, and macrophage infiltrations (**Figure 5C**). Expression abundance analysis revealed a significant positive correlation between C15orf48 and marker genes of these infiltrating cells (**Figure 5E**), especially macrophages (CD11B and CD45) (**Figure 5F**). In addition, C15orf48 was also strongly positively correlated with multiple ICPs in THCA (**Figure 5G**). Moreover, GSVA analysis showed that C15orf48 was significantly correlated with several immune processes, including immune response against tumor cells, cytokine production, and T cell-mediated immune response in THCA (**Figure 5D**).

**Figure 6:**
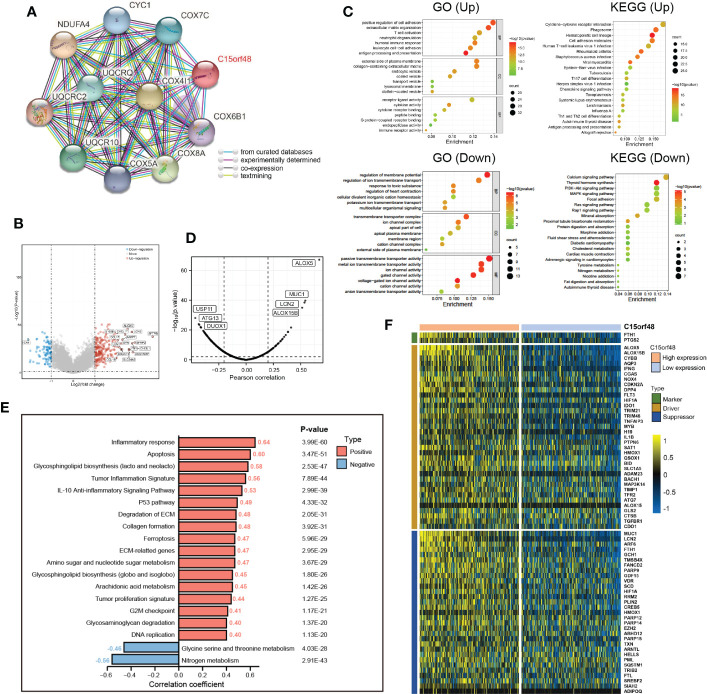
**(A)** The protein interaction network of C15orf48; **(B)** Volcano map of differential genes in C15orf48 high and low expression groups; **(C)** GO and KEGG functional enrichment analysis; **(D)** Correlation between C15orf48 and ferroptosis-related genes in THCA; **(E)** Correlation between C15orf48 and some pathways in THCA; **(F)** Expression of ferroptosis-related genes positively correlated with C15orf48 in THCA.

Furthermore, we explored the functions of C15orf48 in THCA using protein interaction and gene expression data. C15orf48 protein interaction data was obtained from the STRING database (**Figure 6A**). Differential gene expression analysis identified a total of 235 upregulated and 89 downregulated genes in THCA (**Figure 6B**). GO enrichment analysis revealed that the differentially expressed genes (DEGs) were primarily enriched in cell adhesion, transmembrane movement, and immune-related activities, while KEGG enrichment analysis revealed that the DEGs were enriched in PI3K-Akt signaling and cytokine interaction pathways (**Figure 6C**). Pathway analysis showed that C15orf48 had a significant positive correlation with inflammatory response, apoptosis, P53 pathway, ferroptosis, etc. and a significant negative correlation with nitrogen metabolism (**Figure 6E**). Considering that the apoptosis gene set includes pro-apoptotic genes and apoptosis-inhibiting genes, we analyzed the correlation between each apoptosis-related gene and C15orf48. The results showed that C15orf48 was significantly positively correlated with multiple anti-apoptotic factors including baculoviral IAP repeat containing 3 (BIRC3) and B-cell CLL/lymphoma 2 like 1 (BCL2L1), and significantly negatively correlated with pro-apoptotic factors such as caspase 9 (CASP9) and programmed cell death 4 (PDCD4) (**Supplementary Figure 5C**). Considering the high correlation between C15orf48 and ferroptosis, we assessed the correlation between C15orf48 and 484 ferroptosis-related genes, obtained from the FerrDB database (http://www.zhounan.org/ferrdb/current/) (29). The results showed that 322 genes were significantly differentially expressed, among which 68 genes were significantly positively correlated with C15orf48 (35 driver genes, 2 marker genes, and 31 repressor genes) (**Figures 6D, F**). In addition, we analyzed the co-expression of C15orf48 using Co-essentiality (http://coessentiality.net/) (30), and the results revealed the presence of 56 neighborhood genes of C15orf48, which were primarily enriched in lipid and amino acid metabolism (**Supplementary Figures 5A, B**).

**Figure 7:**
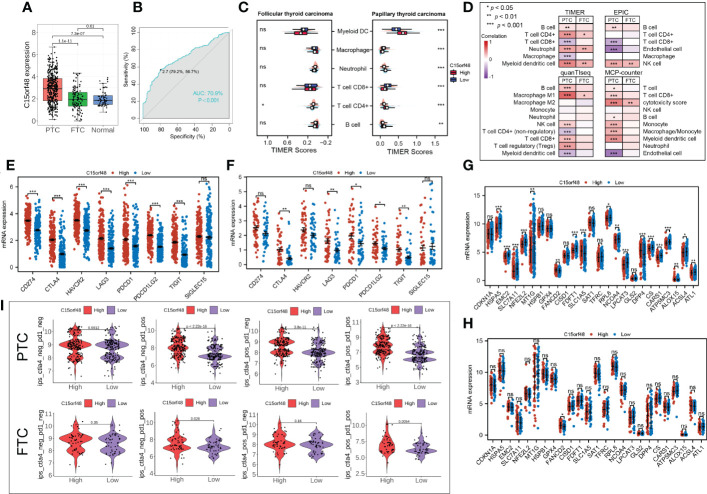
**(A)** Expression levels of C15orf48 in THCA subtypes; **(B)** ROC curves reveal high expression specificity of C15orf48 in PTC subtypes; **(C)** The immune scoring results of different subtypes of THCA (TIMER); **(D)** Correlation between C15orf48 and immune cell infiltration in different THCA isoforms; **(E)** Correlation between C15orf48 and immune checkpoints in PTC subtypes; **(F)** Correlation between C15orf48 and immune checkpoints in FTC subtypes; **(G)** Correlation between C15orf48 and ferroptosis-related genes in PTC subtypes; **(H)** Correlation between C15orf48 and ferroptosis-related genes in FTC subtypes; **(I)** Immunotherapy response results of two subtypes of THCA. *P < 0.05; **P < 0.01; ***P < 0.001, ns, no significance.

Subtype analysis showed that C15orf48 was significantly enriched in PTC. The ROC curve revealed the expression specificity of C15orf48 in PTC subtypes, with the AUC value of 70.9% (*P <*0.0001) (**Figures 7A, B**), suggesting that C15orf48 may serve as a potential biomarker of PTC subtypes. In addition, compared with FTC, PTC subtypes had higher immune scores (**Figure 7C**), and ICI analysis showed that C15orf48 in the PTC group had a significant correlation with various immune cells (**Figure 7D**). The correlation of C15orf48 with ICPs was higher in the PTC group (**Figures 7E, F**), thus compared with the FTC group, the PTC C15orf48 high-expression group benefited more from ICB (ICP blockade) treatment (**Figure 7I**). Furthermore, we assessed the association of C15orf48 with ferroptosis genes in both the subtypes and found a relatively higher correlation between C15orf48 and ferroptosis in the PTC group (**Figures 7G, H**).

### 3.6 Effects of C15orf48 on proliferation, migration, and apoptosis of THCA cells

**Figure 8:**
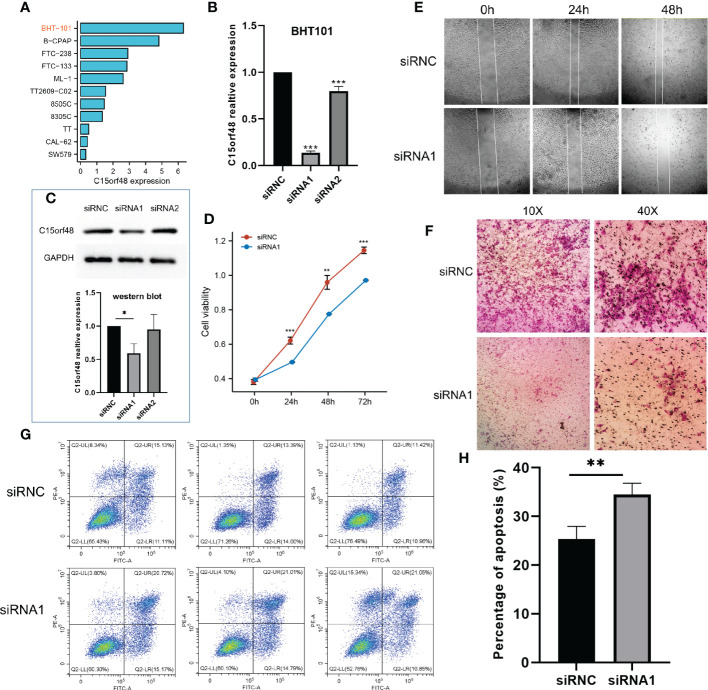
Effect of C15orf48 knockdown on THCA cell line BHT101, all experiments were performed in triplicate. **(A)** Expression levels of C15orf48 in different THCA cell lines; **(B)** RT-PCR verification of the knockout efficiency of C15orf48 in BHT101 cells; **(C)** The knockout efficiency of C15orf48 in BHT101 cells was verified by Western blot, and the figure below shows the statistical difference analysis of three repeated experiments; **(D)** CCK8 assay to analyze the effect of knocking out C15orf48 on cell proliferation; **(E)** Analysis of the effect of knocking out C15orf48 on cell healing ability by cell scratch test; **(F)** Transwell assay to analyze the effect of knocking out C15orf48 on cell migration; **(G)** Analysis of cell apoptosis changes by flow cytometry; **(H)** The percentage of apoptotic cells in the two groups. *P < 0.05; **P < 0.01; ***P < 0.001.

We first analyzed the CCLE data and observed that C15orf48 expression was the highest in BHT101 cells (**Figure 8A**). Therefore, BHT101 cells were selected for subsequent experiments. We transfected BHT101 cells with two siRNA knockout vectors and conducted RT-PCR and western blot analyses. The results revealed that compared with the control group, the expression of mRNA and protein expression in the transfected group were lower, with siRNA1 showing higher knockout efficiency (**Figures 8B, C**). Therefore, siRNA1 was selected for subsequent experiments. The CCK-8 analysis after siRNC and siRNA1 transfection revealed that the proliferation ability of cells was significantly reduced after 24 h of C15orf48 knockout (siRNC: 0.62 ± 0.020, siRNA1: 0.50 ± 0.002) (**Figure 8D**). Additionally, the healing and migration abilities of the BHT101 cells were significantly weakened after C15orf48 knockout, as revealed by the cell scratch and Transwell assays, respectively (**Figures 8E, F**). Lastly, the apoptosis assay showed that the knockdown of C15orf48 significantly increased the rate of apoptosis of BHT101 cells (siRNC: 25.34 ± 2.624, siRNA1: 34.53 ± 2.278) (**Figures 8G, H**).

## 4 Discussion

Mitochondrial dysfunction is a hallmark of immune-mediated inflammatory diseases (31). C15orf48, as part of complex IV of the mitochondrial respiratory chain, is important in the inflammatory response. Clayton et al. demonstrated that the expression of C15orf48 is a conserved response to inflammatory signals and occurs in multiple inflammation-related pathways (18). Significant upregulation of C15orf48 was observed in both rheumatoid arthritis and COVID-19 and was associated with the expression of related macrophage subsets (18). Chronic inflammation is critical for promoting tumor development and drug resistance (32). Specifically, chronic inflammation is associated with immunosuppression. Therefore, it provides a favorable microenvironment for tumor occurrence, development, and metastasis (33). In addition, treatment-induced chronic inflammation contributes to treatment resistance and cancer progression. The inflammatory TME is a key determinant of the efficacy of conventional chemotherapy (radiotherapy and chemotherapy) and immunotherapy (34, 35). However, there is limited information about the role of C15orf48 in tumors.

In our study, we evaluated the pan-cancer expression level of C15orf48 and found that it was significantly upregulated in most tumors, possibly owing to its association with the inflammatory response. Furthermore, the single-cell analysis revealed its immune cell-specific expression in macrophages, suggesting its role in promoting monocyte/macrophage phagocytosis in tumors. Survival analysis showed that C15orf48 was significantly correlated with OS, PFS, DSS, and DFI of multiple cancers, especially glioma. Further univariate and multivariate analyzes revealed that C15orf48 can serve as an independent prognostic factor for glioma. Furthermore, C15orf48 was significantly enriched in malignant gliomas, suggesting its role in promoting the malignant development of gliomas. Altogether, these results illustrate the importance of C15orf48 in tumorigenesis and prognosis. Spisák et al. observed a significant downregulation of C15orf48 methylation in colon cancer tumors (14). Furthermore, analysis of TCGA methylation data revealed the pan-cancer epigenetic changes of C15orf48 and found that the methylation level of C15orf48 was downregulated in multiple cancers, including THCA and kidney cancer. Moreover, we observed a significant negative correlation between C15orf48 methylation levels and mRNA expression in most cancers, suggesting that the methylation level of C15orf48 mediates their abnormal expression, which may play an important role in cancer progression. Furthermore, CNV analysis revealed that the frequency of copy number alterations in the C15orf48 gene was highly heterogeneous. CNV is an important part of genome structural variation, affecting the expression of protein-coding and non-coding genes and the activity of various signaling pathways. More importantly, aberrant methylation of C15orf48 and CNVs leads to poor prognosis in multiple cancers, and it is suggested that epigenetic changes of C15orf48 may promote the progression of some cancers. The TME is critical in the immune response of cancer patients, and the level of ICI is significantly correlated with tumor development (36, 37). The results of the immune analysis showed that C15orf48 was significantly associated with the immune response of THCA, TGCT, LIHC, etc. In addition, C15orf48 was significantly associated with immunotherapy response in several cancers and may serve as a potential target for immunotherapy. Considering that high expression of C15orf48 is associated with higher anticancer immunity but negatively correlated with T cell immunity. Thus, if C15orf48 is targeted in mouse models, increased anticancer immunity but decreased T cell immunity may be observed. In human beings, researchers may observe a similar phenomenon, although the magnitude of the effect may be different. However, this requires further research to understand the potential effects of targeting C15orf48 on both anti-cancer immunity and T cell immunity in human beings.

In China, the incidence of THCA has increased the most in recent years (38). Considering the abnormal expression of C15orf48 in THCA and its strong correlation with the immune response, we focused on analyzing the role of C15orf48 in THCA. C15orf48 was significantly associated with multiple immune modulators, especially some chemokines and MHC molecules. Some chemokines recruit immunosuppressive cells, including macrophages and myeloid-derived suppressor cells, to the TME to create an immunosuppressive but pro-tumor environment, thereby undermining the efficacy of immunotherapies, such as anti-PD1. These results underscore the strong association of C15orf48 with immune responses in THCA. Anti-cancer immune status comprehensively reflects the outcome of immune regulation in the TME. We observed a significant positive correlation between C15orf48 and several steps of the cancer immune cycle. For example, macrophage and monocyte recruitment were significantly increased in the C15orf48 high-expression group, which may be due to a significant increase in macrophage infiltration. In addition, C15orf48 expression was significantly negatively correlated with the recognition of cancer cells by T cells (step 6), which may be due to the significantly high expression of multiple inhibitory ICPs in THCA in the C15orf48 high-expression group. It also suggests that the high expression of C15orf48 may reduce the recognition ability of T cell receptors. The overexpression of inhibitory ICPs, such as PD-1/PD-L1, in the C15orf48 high-expression group, may form a persistent inflammatory TME (39). These results indicate that ICB treatment may be effective for the C15orf48 high-expression group, but not the low-expression group. Pathway analysis showed that C15orf48 was significantly associated with various pathways, such as apoptosis, P53 pathway, and ferroptosis, which are critical in cancer development and immunotherapy (40, 41).

Subtype studies have revealed heterogeneity among the different subtypes in THCA. Our results revealed that C15orf48 was highly expressed in PTC and could potentially serve as a biomarker for PTC. PTC is derived from the acinar cells of the thyroid gland, accounting for more than 80% of THCA, and has a relatively low malignancy, while FTC is more aggressive, with more common distant metastasis and vascular invasion (42–44). Immune scoring reveals specific immune signatures among different subtypes. C15orf48 was involved in several immune responses in PTC and was significantly associated with multiple ICPs and ICI. *In vitro* experiments revealed that C15orf48 knockout significantly reduced the proliferation, migration, and apoptosis abilities of BHT101 cells. Based on these results, we speculate that the C15orf48-related apoptosis may be the mitochondrial/cytochrome c-mediated apoptosis pathway; however, further experimental studies are required to verify this hypothesis.

In conclusion, the results of our study revealed that C15orf48 is a potential tumor prognostic biomarker and immunotherapy target. We found that the pan-cancer epigenetic alterations of C15orf48 are highly heterogeneous and that aberrant methylation and copy number variation of C15orf48 is associated with poor prognosis in several cancers. We also found that C15orf48 was significantly associated with macrophage infiltration and multiple ICPs in THCA and can serve as a potential biomarker for PTC. Lastly, we found that *in vitro* knockdown of C15orf48 reduced the proliferation, migration, and apoptosis abilities of the THCA cell line. Our study still has some limitations. First of all, our immunological research on C15orf48 is only limited to bioinformatics analysis, lacking corresponding laboratory data. Second, the research on THCA is not deep enough, and there is a lack of specific mechanism studies, and more in-depth studies are needed to provide more insights. The findings of our study may help to understand the role of C15orf48 in pan-cancer tumorigenesis and progression, especially in THCA, and provide the basis for further immunotherapy research

## Data availability statement

The original contributions presented in the study are included in the article/**Supplementary Material**. Further inquiries can be directed to the corresponding authors.

## Author contributions

CL and HS conceived the initial research project. QL, HL, and XM performed the statistical analysis and explained the data together with CL. YT and LH assisted CL in completing the cell experiment. CL wrote the first draft of the manuscript. All authors have read and agreed to the published version of the manuscript.
