# Supplementary material for: The prognostic and immune significance of C15orf48 in pan-cancer and its relationship with proliferation and apoptosis of thyroid carcinoma
Source: Front Immunol. 2023 Mar 9;14:1131870. doi: 10.3389/fimmu.2023.1131870 (PMC10033576; doi:10.3389/fimmu.2023.1131870)
Supplement: Supplementary file 1 [file DataSheet_1.docx]

Supplementary Material

The prognostic and immune significance of C15orf48 in pan-cancer and its relationship with proliferation and apoptosis of thyroid carcinoma

Chaolin Li^1*^, Yan Tang^2^, Qin Li^1^, Haiyan Liu^1^, Xiaoying Ma^1^, Liu He^3^, Hao Shi^3*^

*** Correspondence:** Chaolin Li (kone_lcl@foxmail.com); Hao Shi (JNFY_shihao@163.com).

# Supplementary Table

**Supplementary Table 1. 33 kinds of cancer detailed names and sample information**

| **Type** | **Description** | **Tumor samples (TCGA)** | **Normal samples (TCGA+GTEx)** |
| --- | --- | --- | --- |
| ACC | Adrenocortical carcinoma | 79 | 258 |
| BLCA | Bladder Urothelial Carcinoma | 406 | 40 |
| BRCA | Breast invasive carcinoma | 1101 | 572 |
| CESC | Cervical squamous cell carcinoma and endocervical adenocarcinoma | 306 | 22 |
| CHOL | Cholangiocarcinoma | 35 | 9 |
| COAD | Colon adenocarcinoma | 455 | 820 |
| DLBC | Lymphoid Neoplasm Diffuse Large B-cell Lymphoma | 48 | 929 |
| ESCA | Esophageal carcinoma | 163 | 1456 |
| GBM | Glioblastoma multiforme | 153 | 2647 |
| HNSC | Head and Neck squamous cell carcinoma | 504 | 44 |
| KICH | Kidney Chromophobe | 65 | 114 |
| KIRC | Kidney renal clear cell carcinoma | 532 | 161 |
| KIRP | Kidney renal papillary cell carcinoma | 290 | 121 |
| LAML | Acute Myeloid Leukemia | 150 | 0 |
| LGG | Brain Lower Grade Glioma | 513 | 2642 |
| LIHC | Liver hepatocellular carcinoma | 371 | 276 |
| LUAD | Lung adenocarcinoma | 516 | 637 |
| LUSC | Lung squamous cell carcinoma | 501 | 627 |
| MESO | Mesothelioma | 87 | 0 |
| OV | Ovarian serous cystadenocarcinoma | 376 | 180 |
| PAAD | Pancreatic adenocarcinoma | 179 | 332 |
| PCPG | Pheochromocytoma and Paraganglioma | 181 | 3 |
| PRAD | Prostate adenocarcinoma | 498 | 297 |
| READ | Rectum adenocarcinoma | 165 | 789 |
| SARC | Sarcoma | 260 | 2 |
| SKCM | Skin Cutaneous Melanoma | 471 | 1810 |
| STAD | Stomach adenocarcinoma | 375 | 391 |
| TGCT | Testicular Germ Cell Tumors | 134 | 361 |
| THCA | Thyroid carcinoma | 512 | 712 |
| THYM | Thymoma | 120 | 2 |
| UCEC | Uterine Corpus Endometrial Carcinoma | 545 | 177 |
| UCS | Uterine Carcinosarcoma | 57 | 142 |
| UVM | Uveal Melanoma | 80 | 0 |

# Supplementary Figures


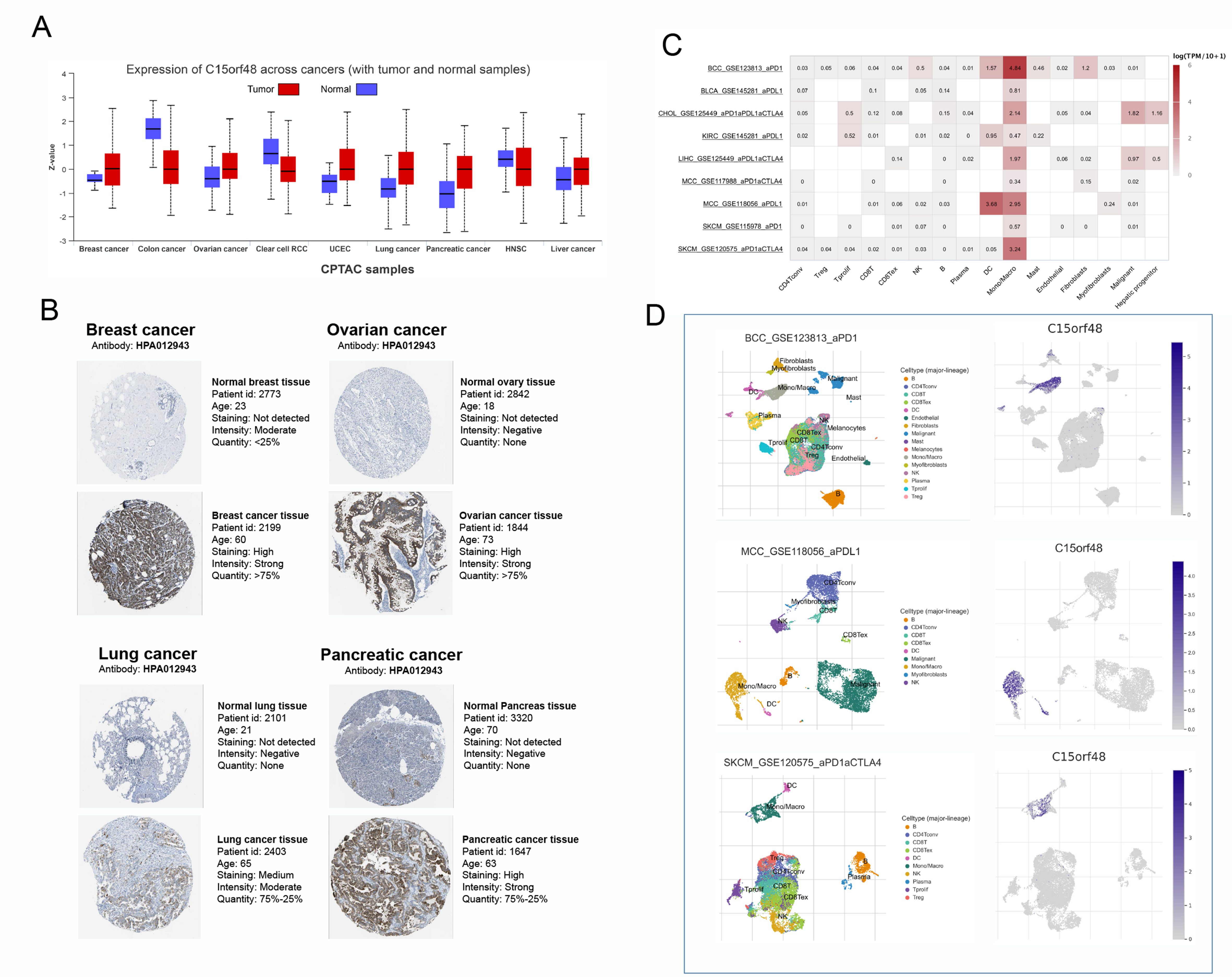


**Supplementary Figure 1.** (A) Pan-cancer analysis of C15orf48 protein expression levels; (B) HPA dataset validates the protein expression of C15orf48 in various cancers; (C) Single-cell expression levels of C15orf48 in immunotherapy datasets (TISCH datasets); (D) Single-cell expression analysis of C15orf48 in BCC (Basal Cell Carcinoma), MCC (Merkel Cell Carcinoma), SKCM (Skin Cutaneous Melanoma) immunotherapy datasets.

**
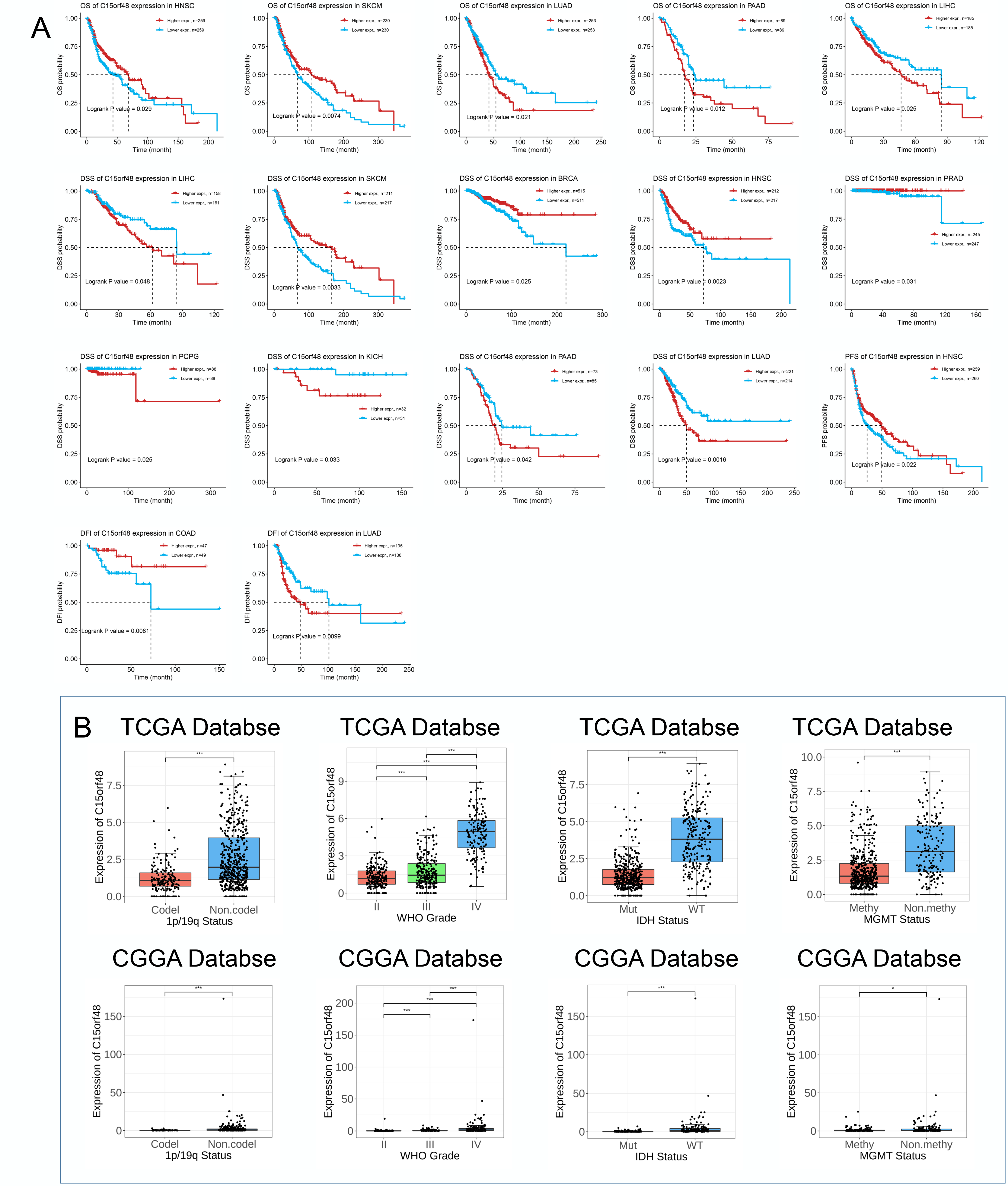
**

**Supplementary Figure 2.** (A) Pan-cancer analysis of the relationship between C15orf48 and prognosis; (B) The relationship between C15orf48 and glioma 1p/19q, Grade, IDH mutation, MGMT status.

**
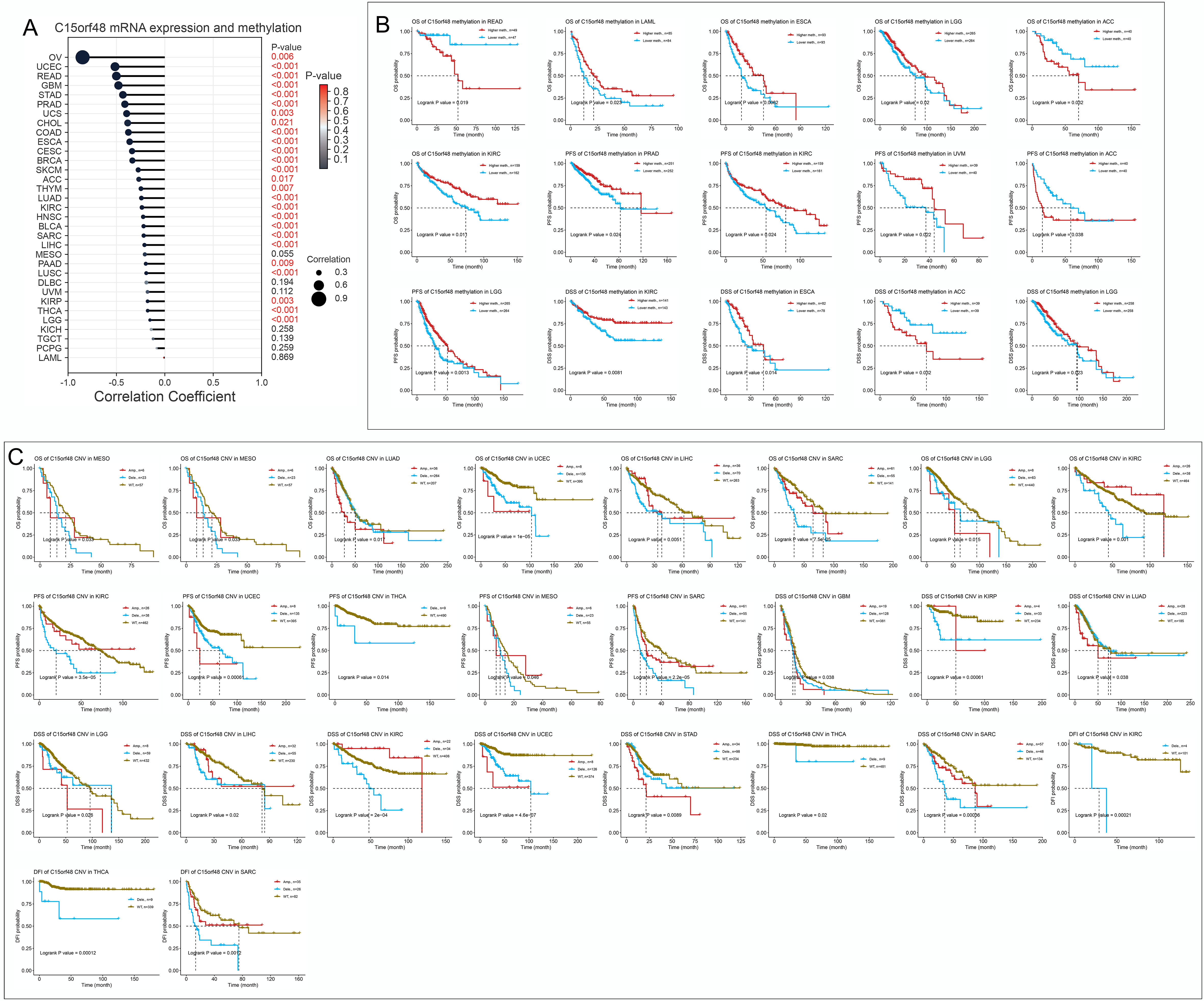
**

**Supplementary Figure 3.** (A) Correlation between mRNA expression and methylation of C15orf48; (B) Correlation between methylation of C15orf48 and prognosis; (C) Correlation between copy number variation and prognosis of C15orf48.

**
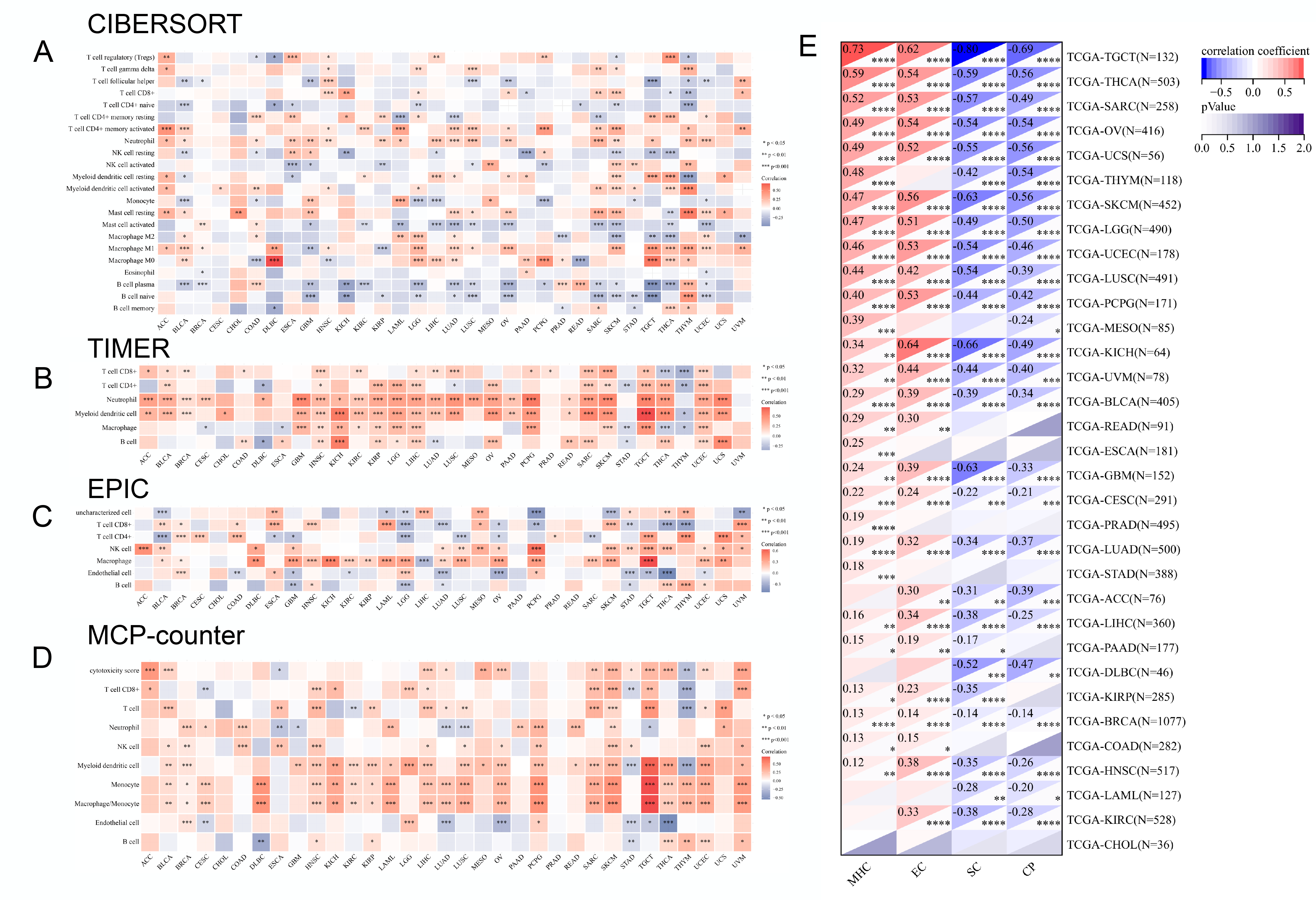
**

**Supplementary Figure 4.** (A) Pan-cancer analysis of the correlation between C15orf48 and immune cell infiltration (CIBERSORT); (B) Pan-cancer analysis of the correlation between C15orf48 and immune cell infiltration (TIMER); (C) Pan-cancer analysis of the correlation between C15orf48 and immune cell infiltration (EPIC); (D) Pan-cancer analysis of the correlation between C15orf48 and immune cell infiltration (MCP-counter); (E) Pan-cancer analysis of the correlation between C15orf48 and immune cell infiltration (IPS).

**
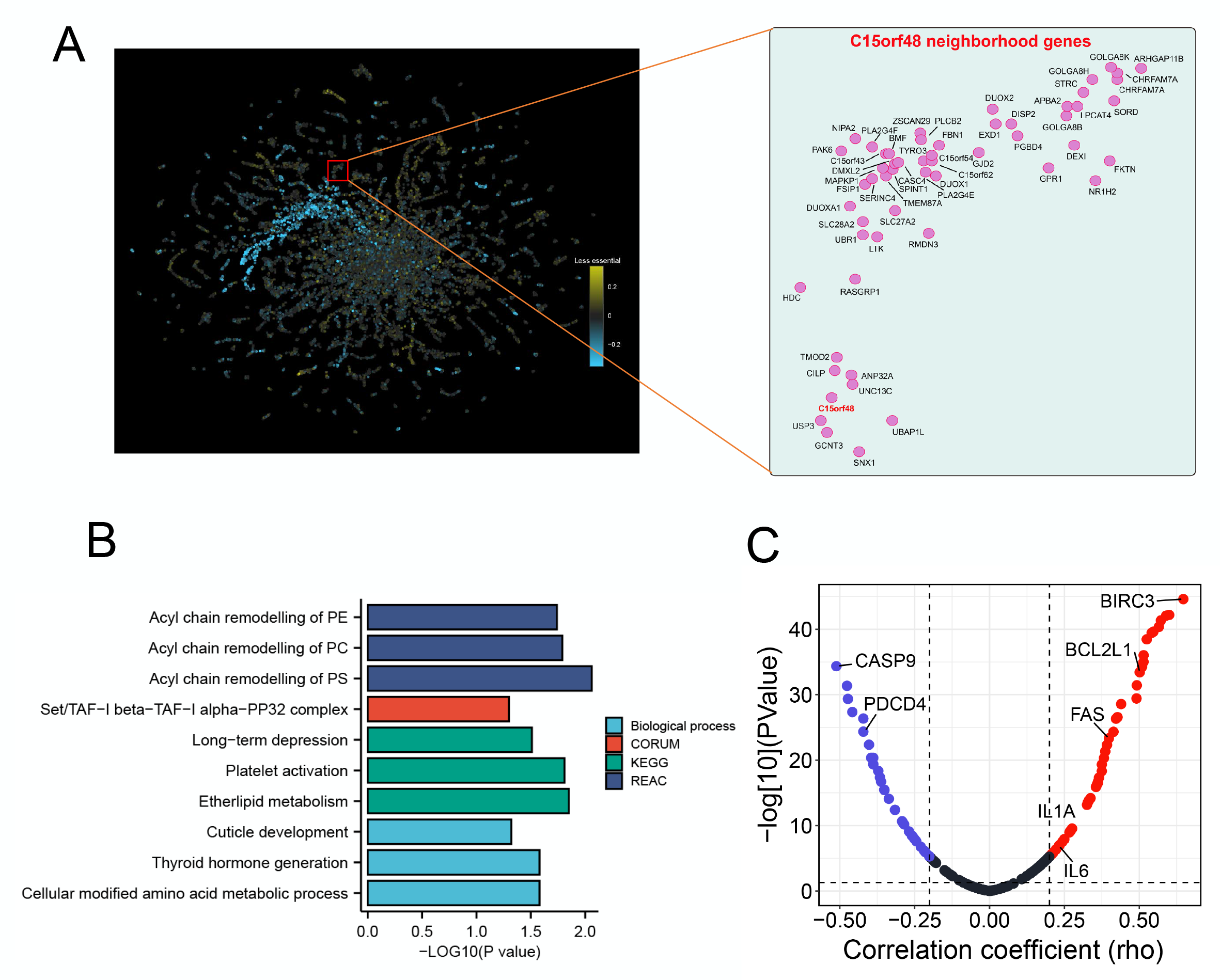
**

**Supplementary Figure 5.** (A) Co-expression analysis network of C15orf48 in THCA; (B) Functional enrichment analysis of C15orf48 neighbor genes; (C) Correlation analysis between C15orf48 and apoptosis-related genes.
